# Distal Biceps Tendon Ruptures: Diagnostic Strategy Through Physical Examination

**DOI:** 10.1177/03635465221129874

**Published:** 2022-11-09

**Authors:** Elisa L. Zwerus, Derek F.P. van Deurzen, Michel P.J. van den Bekerom, Bertram The, Denise Eygendaal

**Affiliations:** † Department of Orthopaedics and Sports Medicine, Erasmus University Medical Centre, Rotterdam, the Netherlands; ‡ Department of Orthopaedic Surgery, Amphia Hospital, Breda, the Netherlands; § Shoulder and Elbow Unit, Department of Orthopaedic Surgery, OLVG, Amsterdam, the Netherlands; ‖ Department of Human Movement Sciences, Vrije Universiteit, Amsterdam, the Netherlands; Investigation performed at Amphia, Breda, OLVG, Amsterdam, the Netherlands

**Keywords:** elbow, tendon, biceps, distal, diagnosis, test

## Abstract

**Background:**

Distinguishing a complete from a partial distal biceps tendon rupture is essential, as a complete rupture may require repair on short notice to restore function, whereas partial ruptures can be treated nonsurgically in most cases. Reliability of physical examination is crucial to determine the right workup and treatment in patients with a distal biceps tendon rupture.

**Purposes:**

The primary aim of this study was to find a (combination of) test(s) that serves best to diagnose a complete rupture with certainty in the acute phase (≤1 month) without missing any complete ruptures. The secondary aims were to determine the best (combination of) test(s) to identify a chronic (>1 month) rupture of the distal biceps tendon and indicate additional imaging in case partial ruptures or tendinitis are suspected.

**Study Design:**

Cohort study (Diagnosis); Level of evidence, 2.

**Methods:**

A total of 86 patients with anterior elbow complaints or suspected distal biceps injury underwent standardized physical examination, including the Hook test, passive forearm pronation test, biceps crease interval (BCI), and biceps crease ratio. Diagnosis was confirmed intraoperatively (68 cases), by magnetic resonance imaging (13 cases), or by ultrasound (5 cases).

**Results:**

A combination of the Hook test and BCI (ie, both tests are positive) was most accurate for both acute and chronic ruptures but with a different purpose. For acute complete ruptures, sensitivity was 94% and specificity was 100%. In chronic cases, specificity was also 100%. Weakness on active supination and palpation of the tendon footprint provided excellent sensitivity of 100% for chronic complete ruptures and partial ruptures, respectively.

**Conclusion:**

The combination of a positive Hook test and BCI serves best to accurately diagnose acute complete ruptures of the distal biceps tendon. Weakness on active supination and pain on palpation of the tendon footprint provide excellent sensitivity for chronic complete ruptures and partial ruptures. Using these tests in all suspected distal biceps ruptures allows a physician to refrain from imaging for a diagnostic purpose in certain cases, to limit treatment delay and thereby provide better treatment outcome, and to avoid hospital and social costs.

Distal biceps tendon ruptures are mostly caused by a sudden extension force on the flexed elbow. Recent literature shows an increasing incidence, while the age of patients with a distal biceps rupture is decreasing.^12^ In the acute setting, it is essential to differentiate between a complete and a partial rupture, as complete ruptures may require surgical fixation on short notice (within 2 to 4 weeks^2,11,20^). Nonoperative management of a complete rupture results in a permanent reduction of flexion and supination strength.^17^ Delayed operative treatment of a complete rupture compromises the ability to perform primary repair due to retraction of the tendon and peritendinous fibrotic scar tissue. This generally necessitates extended reconstructive surgery, which is related to a higher number of surgical complications.^1,11,13,20^ Additional imaging such as magnetic resonance imaging (MRI) or ultrasound is frequently used in case of uncertainty, but physical examination is a less invasive, less costly, and less time-consuming option. MRI is considered the gold standard in diagnosing distal biceps tendon injuries. However, recent research has shown that ultrasound is equally reliable.^4^ A quick diagnosis and surgery is a less important matter in partial ruptures and tendinitis, as there is no retraction of the tendon. However, clinical suspicion based on physical examination in partial ruptures is important since MRI has a low sensitivity (59.1%) but a high specificity (100%).^8^ Accurate physical examination may serve to differentiate between complete and partial ruptures and tendinitis of the distal biceps tendon, leading to optimal treatment decision and indication for additional imaging.

Several retrospective studies have been published on physical examination tests for complete distal biceps ruptures, using MRI and/or surgery as reference test.^5-7,16,18,21^ At the start of our study, no studies were published on partial ruptures or tendinitis. None of the studies on complete ruptures distinguished whether the diagnostic test was performed on acute (≤1 month) or chronic cases. Since local pathology and findings at physical examination may be different, this differentiation is essential as it involves a different treatment regimen. For example, in chronic ruptures, scar tissue may be mistaken for an intact distal biceps and influence diagnostic accuracy of the physical examination. In the acute setting, pain and swelling may also mislead the clinician in his or her assessment of the integrity of the distal biceps.

Furthermore, the diagnostic accuracy of physical examination tests used in previous literature is questionable, since all studies are based on a low number of patients and are retrospective in design, potentially causing spectrum bias. This may result in an overestimation of diagnostic accuracy by means of the positive predictive value (PPV) and negative predictive value (NPV).^19^ Therefore, a prospective study design is preferred to avoid bias and also include patients who do not have the disease of interest.

Subsequent to these requirements, a prospective study was designed with the primary purpose to discover a (combination of) test(s) that serves best to diagnose a complete rupture in the acute phase (≤1 month) without missing any complete ruptures. Our secondary aims were to determine the best (combination of) test(s) to confirm a chronic (>1 month) rupture of the distal biceps tendon and set the indication for additional imaging in cases that are potentially partial ruptures or tendinitis. Ultimately, these data may serve a diagnostic algorithm for clinical use.

## Methods

### Patient Selection

A prospective cohort of consecutive patients with anterior elbow complaints or suspected distal biceps injury was included in our study. Inclusion took place in the outpatient clinics and emergency departments of 2 large teaching hospitals between January 2017 and July 2020. Patients were excluded in case of penetrating trauma or fracture, insufficient knowledge of the Dutch or English language, or significant cognitive impairment. Ethical approval was not required according to the Central Committee on Research Involving Human Subjects, as no additional interventions were performed other than standard care (https://english.ccmo.nl/investigators/legal-framework-for-medical-scientific-research/your-research-is-it-subject-to-the-wmo-or-not).

### Data Collection

Physical examination was performed once on every patient by 4 experienced orthopaedic upper limb surgeons (D.F.B.v.D., M.P.J.v.d.B., B.T., D.E.). The following data were collected using a standard elbow questionnaire: sex, age, hand dominance, affected arm, trauma, and duration of complaints (in months). Physical examination consisted of general elbow examination and specific examination of the distal biceps based on literature.^24^ General examination included carrying angle (normal, valgus, varus), flexion-extension, and supination-pronation elbow range of motion.

#### Index tests

Specific examination consisted of the following items: palpation of the distal biceps footprint on the radial tuberosity, active supination against resistance, the Hook test for integrity of the tendon and for pain,^18^ the passive forearm pronation (PFP) test for integrity and for pain,^5,10^ and the biceps crease interval (BCI) and biceps crease ratio (BCR).^7^ Description of general examination and specific examination tests is provided in Table 1.

**Table 1:** General Examination and Physical Examination Tests

Test	Description
General examination	
Carrying angle (normal/valgus/varus)	
Range of motion (normal or decreased compared with contralateral side)	Flexion-extension
	Supination-pronation
Specific tests	
Hook test^18^	
Patient position	Seated, passive supination forearm, 90° of elbow flexion
Examiner position	Index finger on antecubital fossa
Test	Hook index finger under intact biceps tendon from lateral side
Assessment	No cordlike structure to hook a finger indicates total distal biceps rupture; painful test indicates partial rupture
Passive forearm pronation test^5,10^	
Patient position	Seated, 90° of elbow flexion
Examiner position	Hand on biceps muscle belly, fixate wrist
Test	Palpate biceps muscle belly while pro/supinating forearm passively
Assessment	No proximal excursion of biceps muscle belly in supination and distal migration in pronation indicates total distal biceps rupture; painful test indicates partial rupture
Biceps crease interval (BCI)^7^	
Patient position	Seated, 90° of elbow flexion
Examiner position	Fixate wrist, index finger on antecubital fossa
Test	Passively extend the elbow, supinate forearm; mark flexion crease in antecubital fossa; mark start of the biceps curve; measure distance between marks
Assessment	Absolute BCI value >6 cm indicates complete distal biceps rupture
Biceps crease ratio (BCR)^7^	
Patient position	Seated, 90° of elbow flexion
Examiner position	Fixate wrist, index finger on antecubital fossa
Test	Repeat steps of BCI test on contralateral arm, calculate ratio between BCIs in both arms
Assessment	BCR >1.2 indicates complete distal biceps rupture

#### Reference test

Based on recommendations in the literature, MRI (Ingenia–3T; Phillips Healthcare), ultrasound (GE Logiq E9; GE Healthcare) with a multifrequency linear transducer, and/or confirmative surgery was performed on each patient.^8,15,22^ Surgery was only performed when the patient and surgeon consented on this treatment and not for diagnostic purposes only. Intraoperative findings were considered most accurate and overruled a diagnosis established by imaging. Additional imaging was performed and/or assessed by experienced musculoskeletal radiologists. The examining orthopaedic surgeon (D.F.P.v.D, M.P.J.v.d.B, B.T., D.E.) was blinded for the radiology report and images in case diagnostic imaging was performed before the patients’ visit to the outpatient clinic. Intraoperative assessment was performed by the aforementioned 4 experienced orthopaedic surgeons, who were not blinded for physical examination and/or additional imaging results. A complete rupture was defined as complete loss of distal biceps tendon fibers attached to the radial tuberosity. If a significant part of the tendon fibers remained intact that prevented retraction, the rupture was classified as partial. Cases with a complete rupture of the distal biceps tendon but an intact lacertus fibrosis were also classified as complete distal biceps tendon ruptures. Tendinitis was defined as inflammation around the tendon or bicipitoradial bursa.

### Statistical Analysis

Data analysis was performed using Statistical Package for the Social Sciences 26 (SPSS, Inc). Physical examination tests were analyzed separately and in combination to determine the diagnostic accuracy for the 3 targeted diseases: complete distal biceps tendon rupture, partial distal biceps tendon rupture, and tendinitis of the distal biceps tendon. Acute (≤1 month), chronic (>1 month), and total groups were assessed. Sensitivity, specificity, PPV, NPV, and likelihood ratios (LRs) were calculated using 2 × 2 tables with accompanying 95% CIs. A positive LR (LR+; ie, posttest probability of disease presence) >10 and a negative LR (LR−; ie, posttest probability of disease absence) <0.1 were defined as large.^9^

## Results

A total of 86 consecutive patients met the inclusion criteria and had a complete workup. Nearly half of the patients (n = 42; 49% of the total group) had a complete distal biceps rupture, 29 patients (34%) a partial rupture, 10 (12%) tendinitis, and 5 (6%) another diagnosis.

### Demographics

Most patients were male (n = 78; 91%). Age varied from 24 to 74 years, with a mean of 49.3 years (95% CI, 47.3-51.3). Hand dominance was in line with the general population, and 86% of the patients were right-hand dominant. In 65% of the cases, the dominant side was affected. The median duration of complaints in the total sample was 6.0 months (range, 0-84 months), with 23 patients (27%) seeking treatment within the first month. Among patients with a complete rupture, 21 (50%) sought treatment within the first month, and the other half had chronic complaints. Additional imaging was performed in 68 patients (79%). Of these, 42 had an MRI, 20 had an ultrasound, and 6 patients had both an MRI and ultrasound performed. The remaining 18 patients (21%) underwent surgery without additional diagnostic imaging. In 68 patients (79%), the final diagnosis was confirmed by surgery; 13 (15%) diagnoses were based on MRI findings; and in 5 patients (6%), diagnosis was only established using ultrasound, which confirmed either partial rupture or tendinitis.

### Physical Examination Tests

Pain on palpation of the footprint on the radial tuberosity and weakness with active supination (in 90° of flexion) were sensitive (91% and 95%, respectively) for complete distal biceps ruptures, with weakness with active supination being 100% sensitive in chronic complete ruptures. However, specificity (11% and 21%, respectively), predictive values, and likelihood ratios were low.

The Hook test had moderate sensitivity (71%), but in acute injuries (<1 month), sensitivity was higher (86%). This test was able to correctly reject patients without a complete rupture, with a specificity of 95% overall and 100% in acute cases.

The BCI and BCR showed the same result in all cases. Sensitivity was 81% overall, rising to 86% in acute cases. Specificity was higher in chronic cases (93%) compared with acute injury (50%).

The PFP test demonstrated weaker diagnostic accuracy compared with the Hook test and BCI/BCR. It was less accurate in the acute setting compared with chronic ruptures, with a sensitivity of 74% overall, 67% in acute cases, and 81% in chronic cases. Specificity was 77%, 50%, and 79% for overall, acute, and chronic cases, respectively.

Combining the Hook test and BCI (ie, both tests positive vs none positive), sensitivity was 94% in acute cases, with a specificity and PPV of 100%. For chronic ruptures, this combination was less sensitive (71%), but a specificity and PPV of 100% were observed. Adding the PFP test provided a higher sensitivity in the chronic setting: if 2 of 3 tests were positive (vs none positive), sensitivity was 80%, specificity was 97%, and PPV was 94%. However, to select a patient with a chronic distal biceps tendon rupture for reconstruction surgery, a specificity of 100% was desired, and therefore the combination of the Hook test and BCI was most useful.

Palpation of the footprint on the radial tuberosity and weakness with active supination both had a high sensitivity (100% and 93%, respectively) for partial distal biceps rupture. However, specificity (16% and 10%, respectively), predictive values, and likelihood ratios were low.

A painful Hook test and PFP test showed moderate sensitivity for identifying a partial rupture (76% and 72%, respectively), with low specificity, PPV/NPV, and likelihood ratios. Combining these tests led to a higher sensitivity (up to 85%), but none reached the sensitivity of solely palpation of the footprint. In patients with distal biceps tendinitis, the values were low for all tests.

Diagnostic accuracy of individual tests and combinations is summarized in Table 2.

**Table 2:** Diagnostic Accuracy of Physical Examination Tests (Individually and Combined)^*a*^

Condition	Test	Sens. (%) (95% CI)	Spec. (%) (95% CI)	PPV (%) (95% CI)	NPV (%) (95% CI)	LR+	LR−
Complete rupture (total) (n = 42)	Palpation footprint	90.5 (76.5–96.9)	11.4 (4.3–25.4)	49.4 (37.9–60.9)	55.6 (22.7–84.7)	1.02	0.84
	Active supination	95.2 (82.6–99.2)	20.5 (10.3–35.8)	53.3 (41.5–64.8)	81.8 (47.8–96.8)	1.20	0.23
	Hook test	71.4 (55.2–83.8)	95.5 (83.3–99.2)	93.8 (77.8–98.9)	77.8 (64.1–87.5)	15.71	0.30
	PFP test	73.8 (57.7–85.6)	77.3 (61.8–88.0)	75.6 (59.4–87.1)	75.6 (60.1–86.6)	3.25	0.34
	BCI	81.0 (65.4–90.9)	90.9 (77.4–97.0)	89.5 (74.3–96.6)	83.3 (69.2–92.0)	8.9	0.21
	Combi Hook + BCI (both positive)	82.4 (64.8–92.6)	100 (88.6–100)	100 (85.0–100)	86.4 (72.0–94.3)	Infinity	0.18
	Combi Hook + BCI + PFP (2 out of 3 positive)	87.2 (71.8–95.2)	93.8 (77.8–98.9)	94.4 (80.0–99.0)	85.7 (69.0–94.6)	13.95	0.14
Complete rupture (acute, <1m) (n = 21)	Palpation footprint	90.5 (76.5–96.9)	11.4 (4.3–25.4)	49.4 (37.9–60.9)	55.6 (22.7–84.7)	1.02	0.84
	Active supination	90.5 (68.2–98.3)	50.0 (2.7–97.3)	95.0 (73.1–99.7)	33.3 (1.8–87.5)	1.81	0.19
	Hook test	85.7 (62.6–96.2)	100 (19.8–100)	100 (78.1–100)	40.0 (7.3–83.0)	Infinity	0.14
	PFP test	66.7 (43.1–84.5)	50.0 (2.7–97.3)	93.3 (66.0–99.7)	12.5 (0.7–53.3)	1.33	0.66
	BCI	85.7 (62.6–96.2)	50.0 (2.7–97.3)	94.7 (71.9–99.7)	25.0 (1.3–78.1)	1.71	0.29
	Combi Hook + BCI ( both positive)	94.1 (69.2–99.7)	100 (5.4–100)	100 (75.9–100)	50.0 (2.7–97.3)	Infinity	0.06
	Combi Hook + BCI + PFP (2 out of 3 positive)	94.7 (71.9–99.7)	50.0 (2.7–97.3)	94.7 (71.999.7)	50.0 (2.7–97.3)	1.89	0.11
Complete rupture (chronic, >1m) (n = 21)	Palpation footprint	90.5 (76.5–96.9)	11.4 (4.3–25.4)	49.4 (37.9–60.9)	55.6 (22.7–84.7)	1.02	0.84
	Active supination	100 (80.8–100)	19.0 (9.1–34.6)	38.2 (25.7–52.3)	100 (59.8–100)	1.24	0
	Hook test	57.1 (34.4–77.4)	95.2 (82.6–99.2)	85.7 (56.2–97.5)	81.6 (67.5–90.8)	12	0.45
	PFP test	81.0 (57.4–93.7)	78.6 (62.8–89.2)	65.4 (44.4–82.1)	89.2 (73.6–96.5)	3.78	0.24
	BCI	76.2 (52.5–90.9)	92.9 (79.4–98.1)	84.2 (59.5–95.8)	88.6 (74.6–95.7)	10.67	0.26
	Combi Hook + BCI (both positive)	70.6 (44.0–88.6)	100 (88.3–100)	100 (69.9–100)	88.1 (73.6–95.5)	Infinity	0.29
	Combi Hook + BCI + PFP (2 out of 3 positive)	80.0 (55.7–93.3)	96.7 (80.9–99.8)	94.1 (69.2–99.7)	87.9 (70.9–96.0)	24	0.21
Partial rupture (n = 29)	Palpation footprint	100 (85.4–100)	15.8 (7.9–28.4)	37.7 (27.1–49.5)	100 (62.9–100)	1.19	0
	Active supination	93.1 (75.8–98.8)	10.3 (5.1–19.2)	25.7 (17.9–35.3)	81.8 (47.8–96.8)	1.04	0.67
	Hook test for pain	75.9 (56.1–89.0)	49.1 (35.8–62.6)	43.1 (29.6–57.7)	80.0 (62.5–90.9)	1.49	0.49
	PFP test for pain	72.4 (52.5–86.6)	47.4 (34.2–60.9)	41.1 (27.9–55.8)	77.1 (59.4–89.0)	1.38	0.58
	Combi Hook pain + PFP pain (both positive)	85.0 (61.1–96.0)	47.5 (31.8–63.7)	44.7 (29.0–61.5)	86.4 (64.0–96.4)	1.62	0.32
Tendinitis (n = 10)	Palpation footprint	70.0 (35.4–91.9)	7.9 (3.3–17.0)	9.1 (4.0–18.4)	66.7 (30.9–91.0)	0.76	3.8
	Active supination	60.0 (27.4–86.3)	9.2 (4.1–18.6)	8.0 (3.3–17.2)	63.6 (31.6–87.6)	0.66	4.34
	Hook test for pain	50.0 (20.1–79.9)	39.5 (28.7–51.4)	9.8 (3.7–22.2)	85.7 (69.0–94.6)	0.83	1.27
	PFP test for pain	40.0 (13.7–72.6)	38.2 (27.5–50.1)	7.8 (2.5–19.7)	82.9 (65.7–92.8)	0.65	1.57

a BCI, biceps crease interval; BCR, biceps crease ratio; LR+, positive likelihood ratio; LR-, negative likelihood ratio; PFP, passive forearm pronation; PPV, positive predictive value; NPV, negative predictive value; Sens, sensitivity; Spec., specificity.

## Discussion

This study on the largest prospective series to date in 86 patients demonstrates that a combination of a positive Hook test and BCI is most accurate for both acute and chronic ruptures.

**Figure 1.**
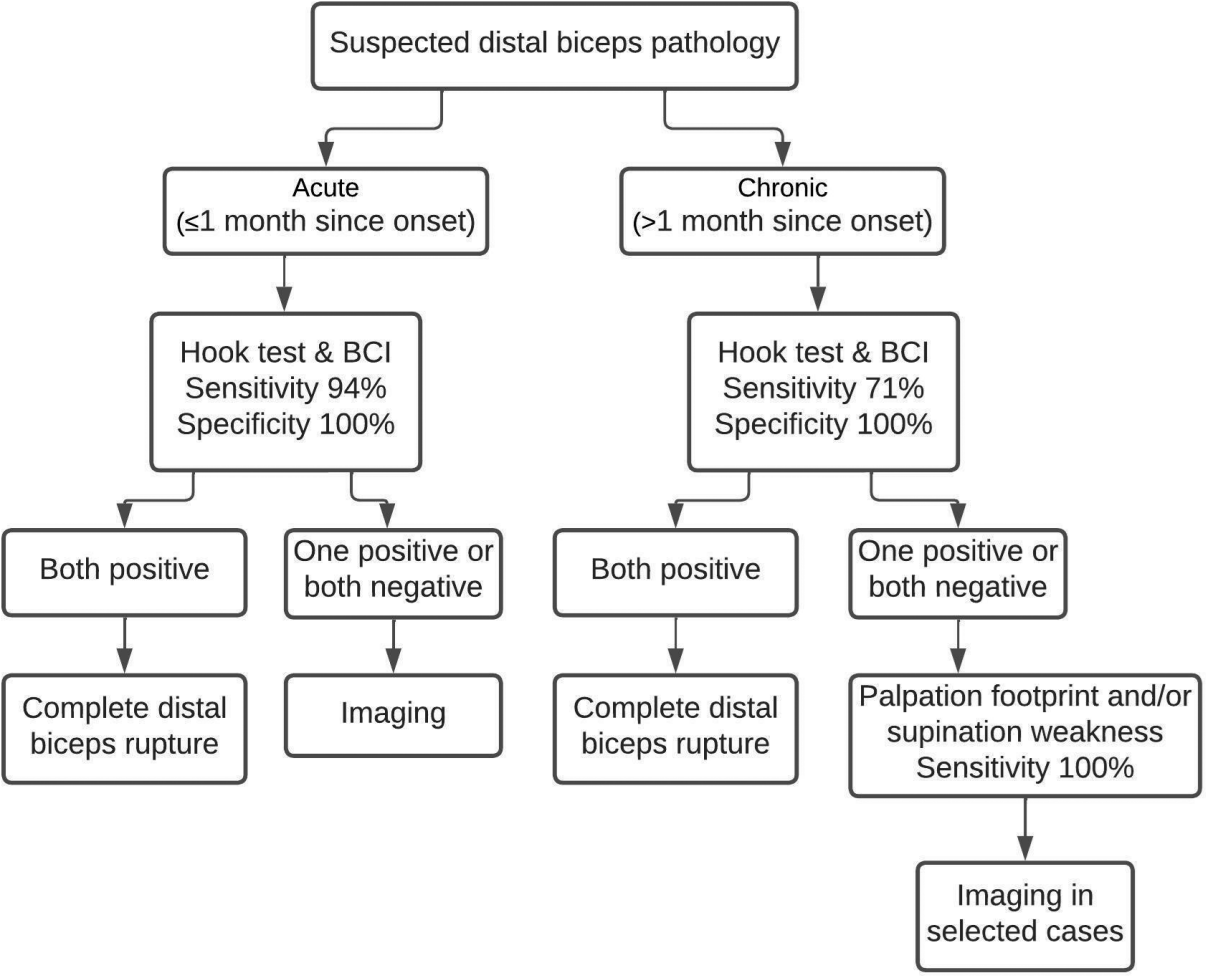
Proposed diagnostic algorithm for suspected distal biceps injuries. BCI, biceps crease interval.

For acute ruptures, a test with well-balanced sensitivity and specificity is essential to rule out anything but a complete rupture with certainty (specificity 100%) without missing anything (sensitivity 94%). For chronic ruptures, it is important to rule out anything but a complete rupture with certainty to decide on operative treatment (specificity 100%). Weakness on active supination and palpation of the footprint provide excellent sensitivity (100%) for chronic complete ruptures and partial ruptures, respectively. For tendinitis, none of the tests provided sufficient diagnostic accuracy to rule in or rule out disease. Using the Hook test and BCI combined in all suspected distal biceps ruptures allows physicians to refrain from imaging for the purpose of diagnosing in certain cases, to limit treatment delay and thereby provide better treatment outcome, and to avoid hospital and social costs. On the basis of our findings, we propose a diagnostic algorithm in Figure 1.

In previous work, we assessed the methodologic quality of all studies on distal biceps pathology published before 2016, identifying bias and applicability concerns.^24^ These concerns are mainly caused by patient selection: half of the studies were case-control designs,^7,16,18^ and all studies had a high prevalence of disease (73%-100%) with a low number of patients (5-48). Recently, a study investigated the sensitivity of the Hook test in a larger sample (202 cases) with a lower prevalence of complete ruptures. However, a retrospective design was used, potentially introducing selection bias.^14^ Also, most studies focused on individual tests for (acute) complete distal biceps tendon ruptures rather than a combination of tests or other entities such as partial ruptures, chronic ruptures, or tendinitis.

The Hook test, PFP test, BCI/BCR, biceps squeeze test, and supination-pronation test were investigated for individual use and showed a sensitivity ranging from 81% to 100% and a specificity from 50% to 100%.^7,14,16,18,21^ One study tested a combination of tests; the Hook test, PFP test, and BCI combined reached a sensitivity of 100% and a specificity of 50%.^5^ In comparison with our results, previous literature on individual tests for complete distal biceps ruptures showed similar diagnostic accuracy. For the combination of tests, the sensitivity in the combinations we investigated was slightly lower: 94% and 80% for acute and chronic ruptures, respectively, in our study, compared with 100% overall in the study by Devereaux and ElMaraghy.^5^ For specificity, however, our sample reached 100% and 97% for acute and chronic ruptures, respectively, compared with 50% by Devereaux and ElMaraghy. However, a direct comparison cannot be made since none of the previous studies differentiated diagnostic accuracy of tests between acute and chronic ruptures.

In all studies, either MRI and/or intraoperative findings were used as a reference test. MRI is the gold standard to diagnose a complete distal biceps tendon rupture, but recent research has shown that ultrasound is equally reliable.^4^ The sensitivity and specificity of MRI for complete tears are 100% and 82.8%, respectively.^8^ Recent literature reported no significant differences in sensitivity and specificity in detecting partial distal biceps injuries when the flexed abducted supinated (FABS) view MRI and standard MRI were compared.^22^ Ultrasound was found equally accurate in cases of complete and high-grade partial distal biceps tendon ruptures.^4^ For partial tears, the overall accuracy rate of MRI (66.7%) was the same as ultrasound, but MRI had a low sensitivity (59.1%) but a high specificity (100%).^8,15^

The main strengths of our study are provided by the fact that it is the largest prospectively collected sample of patients with distal biceps pathology, including both acute and chronic, complete and partial ruptures, as well as tendinitis and other anterior elbow complaints. Using a combination of tests provided even more reliable results, with the ability to more accurately discern between acute and chronic complete ruptures. Therefore, we believe that our results are applicable and valuable for clinical practice.

The distal biceps tendon rupture is an increasingly popular topic for research. During the inclusion period of our study, 2 novel tests for partial ruptures and tendinitis were published. Shim and Strauch^23^ presented a novel clinical test for partial tears of the distal biceps tendon, the TILT sign. This test, however, does not add to our study since it is performed exactly the same way as we performed palpation of the radial tuberosity. Caekebeke et al^3^ introduced the biceps provocation test for partial ruptures and tendinitis, with a sensitivity and specificity of both 100%. This test includes 2 parts: resisted flexion of the elbow with the forearm supinated and with the forearm pronated. The generalizability of this test is questionable since the authors performed the test only on patients with a partial rupture (and healthy participants) and not on patients with a complete rupture. To use this test to differentiate between complete and partial ruptures, further research is needed.

Furthermore, we did not include the supination-pronation test because the only difference between this test and the PFP test is the active motion of the patient (without testing the strength) versus passive motion.^16^ Both tests were designed to test the integrity of the tendon and accompanying muscle belly, so in our experience, active motion has limited added value. However, we incorporated testing the supination strength against resistance to test active motion and strength.

Another limitation is the fact that we did not perform inter- or intrarater reliability. ElMaraghy et al^7^ investigated the interrater reliability for the BCI test, observing an intraclass correlation coefficient of 0.79. For all other tests, no inter- or intrarater reliability was available in the literature, leaving this subject for further research.

## Conclusion

The combination of a positive Hook test and BCI is highly sensitive (94%) and specific (100%) to diagnose acute, complete ruptures of the distal biceps tendon. A similar high specificity (100%) was found for identifying chronic rupture of the distal biceps when the combination of these tests was performed. Weakness on active supination and pain on palpation of the tendon footprint provide excellent sensitivity (100%) for chronic complete ruptures and partial ruptures, but physical examination tests are not accurate enough to confirm either partial rupture or tendinitis, leaving an indication for additional diagnostic imaging in these cases.
